# The relationships between systemic cytokine profiles and inflammatory markers in colorectal cancer and the prognostic significance of these parameters

**DOI:** 10.1038/s41416-020-0924-5

**Published:** 2020-06-03

**Authors:** Ji Won Park, Hee Jin Chang, Hyun Yang Yeo, Nayoung Han, Byung Chang Kim, Sun-Young Kong, Jeongseon Kim, Jae Hwan Oh

**Affiliations:** 1 Department of Surgery and Cancer Research Institute, Seoul National University College of Medicine, Seoul, Republic of Korea; 2 Center for Colorectal Cancer, Research Institute and Hospital, National Cancer Center, Goyang, Republic of Korea; 3 Divison of Precision Medicine, Research Institute, National Cancer Center, Goyang, Republic of Korea; 4 Department of Pathology, Research Institute and Hospital, National Cancer Center, Goyang, Republic of Korea; 5 Department of Laboratory Medicine & Genetic Counseling Clinics, Research Institute and Hospital, National Cancer Center, Goyang, Republic of Korea; 6 Department of Cancer Biomedical Science, Graduate School of Cancer Science and Policy, National Cancer Center, Goyang, Republic of Korea

**Keywords:** Colorectal cancer, Tumour biomarkers

## Abstract

**Background:**

Immunomodulatory cytokines and systemic inflammatory markers are important during cancer development and progression. This study investigated the association and prognostic impact of systemic cytokine profiles and inflammatory markers in colorectal cancer (CRC).

**Methods:**

Interleukin (IL)-1β, IL-6, IL-8, IL-9, IL-10, tumour necrosis factor (TNF)-α and vascular endothelial growth factor (VEGF) serum levels were measured using multiplex bead assays in CRC patients. Data on systemic inflammatory markers, such as the modified Glasgow prognostic score (mGPS), the neutrophil-to-lymphocyte ratio (NLR), platelet-to-lymphocyte ratio (PLR), lymphocyte-to-monocyte ratio (LMR), prognostic nutritional index (PNI) and fibrinogen, were collected. Survival analysis was performed to identify factors associated with progression-free survival (PFS) and overall survival (OS).

**Results:**

There were moderate-to-strong correlations within serum cytokines, as well as within systemic inflammatory markers, whereas the associations between serum cytokines and systemic inflammatory markers were generally weak. IL-8 and the LMR were independent significant prognostic factors for PFS and OS. The low IL-8 and high LMR group had the best survival (both PFS and OS) of all groups.

**Conclusions:**

Systemic cytokine profiles and inflammatory markers have relatively weak intergroup correlations. A composite classification of systemic cytokine profiles and inflammatory markers has an enhanced prognostic value in CRC.

## Background

Tumour pathologic characteristics are associated with prognosis in colorectal cancer (CRC). However, these characteristics alone do not accurately predict the survival outcomes of patients. Tumour-associated inflammation can also determine the prognosis of patients. This type of inflammation is known to occur as a local immune response and as systemic inflammation.^1^ As a part of the local immune response, the composition of tumour-infiltrating lymphocytes in the tumour microenvironment is correlated with prognosis in CRC.^2^ As a part of systemic inflammation, circulating cytokines or systemic inflammatory markers have been suggested as prognostic markers in CRC.^3,4^

Classic inflammatory cytokines, such as interleukin (IL)-1β, IL-6 and tumour necrosis factor (TNF)-α, activate the NF-κB and STAT3 signalling pathways, and induce the expression of genes that promote the invasion of cancer cells and angiogenesis.^5^ Several cytokines, especially IL-6, have been evaluated and found to be associated with survival in CRC.^6^ However, the association between these cytokines and outcomes was not consistent in previous reports. As an inflammatory chemokine, IL-8 induces the proliferation and migration of CRC cells by promoting neutrophil chemotaxis and angiogenesis.^7^ IL-8 has been suggested as a diagnostic marker and a prognostic factor for CRC.^8^ As a cytokine associated with a variety of inflammatory and autoimmune diseases, IL-9 may have a dual role in CRC progression.^9^ Vascular endothelial growth factor (VEGF) promotes tumour angiogenesis, and is the most potent angiogenic growth factor. High serum VEGF levels were associated with poor survival in CRC.^10,11^

Systemic inflammatory markers have been introduced as an integrative method to measure systemic immunity.^12^ These markers include inflammation-associated cell enumeration or serum inflammatory markers, such as C-reactive protein (CRP).^13^ Inflammatory markers, such as the neutrophil-to-lymphocyte ratio (NLR), platelet-to-lymphocyte ratio (PLR) and lymphocyte-to-monocyte ratio (LMR), have been shown to be practical metrics for determining the prognosis in CRC.^14–16^

Tumour-associated inflammation and systemic inflammatory responses have emerged as critical components that govern the clinical outcomes of CRC. However, little is known about the interrelationships and clinical impact of systemic cytokine profiles and inflammatory markers in CRC. This study aimed to investigate the association and prognostic impact of systemic cytokine profiles and inflammatory markers in CRC patients. In addition, the authors evaluated the prognostic value of the composite stratification of systemic cytokine profiles and inflammatory markers for predicting survival in CRC.

## Methods

### Study population and blood collection

The study population consisted of patients with CRC who underwent surgery for primary CRC at the National Cancer Center, Korea. Participants in this study were prospectively recruited for blood sampling between March 2009 and September 2010. Patients who were older than 18 years were eligible. Patients with a previous or synchronous second primary malignancy and a previous history of colorectal surgery for CRC were excluded.

Approximately 5–10 ml of blood was collected from eligible patients before surgery and after informed consent was obtained. Serum separator collection tubes were used for collecting blood, and were centrifuged at 2000 rpm for 10 min at room temperature. The serum was extracted, dispensed into aliquots in polypropylene tubes and stored at –70 °C. Each serum sample was assigned a unique identifier number to conceal the patient information.

### Cytokine analysis and systemic inflammatory markers

Cytokines were assayed using a multiplex bead immunoassay system (Procarta Cytokine Assay Kit, Affymetrix eBioscience, Santa Clara, CA) according to the manufacturer’s instructions in June 2012. Following incubation with multiple antibody-coated microbeads, multiple protein targets were quantified with a multi-analyte profiling technology by using a Luminex 100 (Luminex 100, Luminex Corporation, Austin, TX) and Bio-Plex software (Bio-Plex, Bio-Rad Laboratories Incorporated, Hercules, CA). The following seven cytokines, which are associated with immune cell-mediated inflammation and angiogenesis, were measured: proinflammatory cytokines (IL-1β, IL-6 and TNF-α), inflammation-associated cytokines (IL-8 and IL-9), an anti-inflammatory cytokine (IL-10) and an angiogenetic cytokine (VEGF-A). The assays were performed blinded to the clinical outcomes.

Clinicopathological data and preoperative laboratory results (including the neutrophil, lymphocyte, platelet and monocyte counts, fibrinogen, albumin and CRP levels) were collected. The modified Glasgow prognostic score (mGPS), NLR, PLR, LMR, prognostic nutritional index (PNI) and fibrinogen were assessed as systemic inflammatory markers.^13–18^ The mGPS was calculated based on the serum concentrations of CRP and albumin.^13^ Patients who had normal albumin (>3.5 g/dl) and CRP (< 1.0 mg/dl) levels were assigned a score of 0. Patients with only elevated CRP (> 1.0 mg/dl) were given a score of 1. Those with low albumin (<3.5 g/dl) and high CRP (> 1.0 mg/dl) levels were given a score of 2. The NLR was defined as the absolute neutrophil count divided by the absolute lymphocyte count. The PLR was defined as the absolute platelet count divided by the absolute lymphocyte count. The LMR was defined as the absolute lymphocyte count divided by the absolute monocyte count. The PNI was determined using the following formula: albumin (g/l) + (5 × total lymphocyte count × 10^3^/µl).^19^

### Immunohistochemistry for molecular subtype classification

We classified 138 patients into consensus molecular subtypes (CMS) using immunohistochemistry according to Trinh’s protocol.^20^ Tissue microarray blocks were previously constructed from 316 CRC cases resected in the National Cancer Center, Korea, from 2009 to 2010, and 138 out of 316 cases were matched with this study population. The blocks consisted of each representative tissue core (2 mm in diameter) taken from formalin-fixed paraffin-embedded CRC tissues.

Immunohistochemical staining was performed using a BenchMark XT automated slide stainer (Ventana Medical Systems, Inc., Tucson, AZ, USA), by using anti-CDX2 (1:50, Cell Marque, EPR276RY), anti-ZEB1 (1:1000, Sigma, HPA027524) and anti-HTR2B (1:500, Sigma, HPA012867) antibodies. Antigen retrieval was performed with cell-conditioning solution 1/EDTA (pH 8.0) for 30 min at 98 °C using the BenchMark staining module (Ventana Medical Systems, Inc.). Slides were counterstained with haematoxylin II and Bluing Reagent (cat. no. 760-2037, Ventana Medical Systems, Inc.) for 4 min at room temperature. For the negative control, tissue sections were incubated with Tris-buffered saline alone without the primary antibody.

Immunohistochemical expression was evaluated by two pathologists (HJC and NYH). CDX2 and HTR2B expression was quantitatively evaluated using a double-scoring system by estimating the staining intensity and percentage of stained cancer cells. The staining intensity was classified as 1 (weak), 2 (moderate) or 3 (strong). Immunoreactivity was scored as 0–300 by multiplying the staining intensity by the percentage of cells stained. ZEB1 expression was evaluated by estimating the percentages of stromal nuclear expression within the entire tumour core, since the staining intensity of ZEB1 was relatively even (moderate) in the entire cases. Based on the mean values for expression of each marker, the staining results were classified into ‘low’ or ‘high’ expression. Epithelial–mesenchymal transition (EMT) phenotype was defined as ‘low CDX2’ and ‘high ZEB1 and /or HTR2B’.

Microsatellite-instability phenotype was determined based on the results of immunohistochemistry for four mismatch repair proteins (MLH1, PMS2, MSH2 and MSH6). The antibodies were anti-MLH1 (Ready-to-use, Ventana, M1), anti-MSH2 (1:300, BD Pharmingen, G219-1129), anti-MSH6 (1:300, BD Pharmingen, 44/MSH6) and anti-PMS2 (1:80, BD Pharmingen, A16-4). Loss of mismatch repair proteins was given if there was a distinct loss of nuclear staining in tumour cells, while normal stroma and lymphocytes showed strong nuclear staining in the same areas, thus excluding artefact and/or staining failure. Microsatellite instability was assigned in the case of the loss of any mismatch repair protein.

'Immune' (CMS1) subtype was a microsatellite-instable case. EMT phenotype was classified into ‘mesenchymal' (CMS4) subtype, and the remaining cases were classified into ‘epithelial' (CMS2/CMS3) subtype.

### Immune score analysis

To evaluate immune score in the tumour centre and invasive front, the whole tumour section was necessary, and 311 cases were available for the analysis. Immune score was evaluated by image analysis for CD3- and CD8-expressing lymphocytes in the tumour centre and invasive front, as described previously.^21^ Briefly, immunohistochemical stain was performed by using anti-CD3 (Ready-to-use, Ventana, 2GV6) and anti-CD8 (Ready-to-use, Ventana, SP57) antibodies, and the immunostained slides were scanned on an Aperio ScanScope CS instrument (Aperio Technologies, Inc.). CD3 + and CD8 + lymphocytes were automatically counted by the Nuclear v9 algorithm of ImageScope^TM^ (Aperio Technologies, Inc.) image analysis system. We estimated four densities (number of positive cells per mm^2^) of CD3 + and CD8 + lymphocytes in the tumour centre and invasive front, respectively. If the density of both CD3^+^ and CD8^+^ T cells was elevated (higher than median) in both the centre and front, a high immune score was given.

### DNA isolation

DNA was isolated from four 5-µm slices of formalin-fixed, paraffin-embedded (FFPE) tissues using the QIAamp DNA FFPE Tissue kit (Qiagen, Hilden, Germany) with the following amendments: samples were deparaffinised for 5 min with 20 mL of xylene (Merck KGaA, Darmstadt, Germany). Digestion steps were performed in double volumes, in that protease K digestion was performed in 200 µL of ATL buffer, using 20 µL of Protease K incubation at 65 °C for 50 min, followed by a heating step of 98 °C for 1 h, 30 min. RNA digestion was carried out, using 2 µL of RNase A, then 200 µL of AL buffer was added and 200 µL of ethanol (100%) was vortexed and loaded into columns in two steps. DNA was eluted in 20 µL of nuclease-free water and quantified using a Nanodrop 2000 spectrophotometer (ThermoScientific, MA, USA).

### Mutation analysis

To assess the relation between systemic markers and tumour molecular alteration, we performed somatic mutation analysis of seven genes associated with colorectal cancer (*KRAS, BRAF, NRAS, APC, PIK3CA*, *PTEN* and *TP53*).

Mutation detection was performed using iPLEX^®^ Pro reagent on MassARRAY^®^ System (Agena Bioscience, CA). The 53-hotspot target mutation sites of the following genes: APC, BRAF, KRAS, NRAS, PIK3CA, PTEN and TP53 for colon cancer were designed (Supplementary Table 1). About 10 ng of genomic DNA was amplified using HotStarTaq polymerase (Qiagen, Valencia, CA), 100 nm of primers and 0.5 mM dNTPs (Invitrogen, Inc.) on 9700 thermal cycler (Thermoscientific, MA, USA). PCR products were treated with SAP (Shrimp Alkaline Phosphatase) enzyme. Single-base extension reaction was performed followed SAP treatment. The final product was cleaned with resin, and 16-nl product was transferred to a spectrochip using Nanodispenser RS 1000 (Agena Bioscience, CA). Finally, one nucleotide difference was detected using MALDI-TOF, and allele frequency was calculated using TYPER v4.0 software. This work was supported by the Genomics Core Facility in the National Cancer Center Korea.

### Follow-up

After surgery, adjuvant chemotherapy was performed for patients with stage II with high risk, stage III and stage IV. The patients were followed up every 3 or 6 months for 5 years, and then every year thereafter. Physical examinations, chest X-ray, serum carcinoembryonic antigen (CEA) levels and abdominopelvic computed tomography were performed every 3 or 6 months. A colonoscopy was performed at 1 year after surgery, and every 2 years thereafter. Recurrence was detected by imaging tests, a biopsy or a combination of these methods.

### Statistical analysis

The Reporting Recommendations for Tumor Marker Prognostic Studies (REMARK) criteria were taken into account in the study reporting (Supplementary Table 2).^22^ For comparison of the variables, Chi test, Mann–Whitney *U* test or Kruskal–Wallis test were used according to the types of variables. Spearman’s correlation tests were used to assess the pairwise relationship among cytokines and systemic inflammatory markers. The cut-off values of each cytokine and fibrinogen were determined by the median value. The cut-off values of systemic inflammatory markers were published in previous studies (NLR: 5, PLR: 150, LMR: 3 and PNI: 45).^14,15,23,24^ PFS was defined as the time to the recurrence or progression of CRC. OS was defined as the time to death of any cause. The log-rank test was used to compare the survival of patients with these variables. Using a forward selection method, Cox proportional hazard analysis was used for multivariable survival analysis, which included variables with values of *P* < 0.1. Although no formal sample-size calculation was conducted in advance, the number of events (nearly 80 deaths and 120 progressions) compared with the number of Cox model variables (5 or 8) implied that the ‘a minimum of 10 events per predictor' rule was exceeded, indicating the accuracy and precision of the regression estimates.^25^ All analyses were performed using SPSS version 22.0 (SPSS Inc., Chicago, IL, USA). A value of *P* < 0.05 was considered statistically significant.

## Results

Among 870 patients who underwent surgery for CRC during the enrolment period, 168 patients with a previous or synchronous malignancy and 7 patients who were younger than 18 years were excluded. In the group of eligible patients, a total of 400 patients agreed to participate and were included in this study.

The clinicopathological characteristics of patients are shown in Table 1. The median age of the patients was 62 years [interquartile range (IQR): 53–69 years]. The median body mass index (BMI) was 23.6 kg/m^2^ (IQR: 21.6–25.6 kg/m^2^), and the median CEA level was 3.2 ng/ml (IQR: 2.0–7.0 ng/ml).

**Table 1:** Clinicopathological characteristics of patients.

Characteristics	*N* = 400
*Age*	
≤60 years	186 (46.5%)
>60 years	214 (53.5%)
*Gender*	
Male	244 (61.0%)
Female	156 (39.0%)
Body mass index^*^	
≤25 kg/m^2^	279 (69.9%)
>25 kg/m^2^	120 (30.1%)
*Location*	
Colon	211 (52.8%)
Rectum	189 (47.3%)
*Serum CEA*	
≤5 ng/mL	261 (65.3%)
>5 ng/mL	139 (34.8%)
*Histologic grade*^†^	
Low	376 (94.5%)
High	22 (5.5%)
*Stage*	
1	105 (26.3%)
2	110 (27.5%)
3	149 (37.3%)
4	36 (9.0%)
*Lymphovascular invasion*^¶^	
No	74 (19.2%)
Yes	311 (80.3%)
*Adjuvant chemotherapy*	
No	135 (33.8%)
Yes	265 (66.3%)
*Neoadjuvant chemoradiotherapy*	
No	361 (90.2%)
Yes	39 (9.8%)

*CEA* carcinoembryonic antigen.

^*^399, ^†^398 and ^¶^385 were available.

**Fig. 1:**
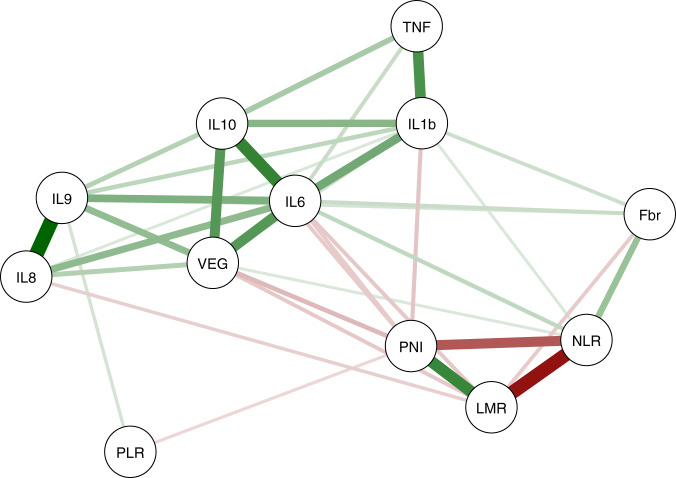
Pairwise correlation graph between systemic cytokine profiles and inflammatory markers. Nodes represent systemic cytokine profiles or inflammatory markers, and lines represent their pairwise correlations. Only significant (*P* < 0.05) correlations are shown. Line thickness indicates the strength of Spearman’s correlation. Green represents positive correlations, and red represents negative correlations. IL interleukin, TNF tumour necrosis factor, VEGF vascular endothelial growth factor, NLR neutrophil-to-lymphocyte ratio, PLR platelet-to-lymphocyte ratio, LMR lymphocyte-to-monocyte ratio, PNI prognostic nutritional index, Fbr fibrinogen.

The correlations within cytokines were moderate to strong for some cytokines (Fig. 1; Supplementary Table 3; IL-8 and IL-9: Spearman’s correlation coefficient [ρ] = 0.71, IL-6 and IL-10: ρ = 0.57 and IL-6 and VEGF-A: ρ = 0.47). There was also a moderate-to-strong correlation within some systemic inflammatory markers (NLR and LMR: ρ = −0.66, LMR and PNI: ρ = 0.55 and NLR and PNI: ρ = −0.47). However, the associations between serum cytokines and systemic inflammatory markers were generally weak (Fig. 1, −0.19 ≤ ρ ≤ 0.17).

The levels of cytokines and systemic inflammatory markers were not significantly different among immune (CMS1), epithelial (CMS2/3) and mesenchymal (CMS4) subtypes (Supplementary Table 4). *KRAS* mutation was related with low NLR, high LMR and high PNI (Supplementary Table 5). *PIK3CA* mutation was associated with high LNR and high PLR. *TP53* mutation was related with low PLR, high LMR and high PNI. There were no significant systemic markers associated with *BRAF, NRAS, APC* and *PTEN*. Within tumour-infiltrating lymphocytes, there were strong correlations (Supplementary Fig. 1). There were only weak correlations between CD3^+^/CD8^+^ T-cell counts and systemic inflammatory markers. However, there was no significant correlation between CD3^+^/CD8^+^ T-cell counts and cytokines. High immune score was associated with low NLR, high PNI and low fibrinogen (*P* = 0.005, *P* = 0.042 and *P* = 0.011, respectively, Supplementary Table 6). Microsatellite-instable tumour was associated with high NLR, high PLR and low PNI (*P* = 0.033, *P* = 0.041 and *P* = 0.015, respectively, Supplementary Table 6). However, cytokines were not related to tumour molecular alteration, immune score and microsatellite instability.

For survival analysis, patients were divided into low and high groups according to median values of cytokines and fibrinogen (Table 2). The median time of follow-up was 63.7 months (IQR: 49.0–84.0 months). In univariate survival analysis, IL-6, IL-8, VEGF-A, the mGPS, NLR, LMR and fibrinogen were significantly associated with PFS and OS (Table 3). In multivariable analysis for PFS using variables with values of *P* < 0.1, IL-8 (hazard ratio [HR] = 1.82, 95% confidence interval [CI]: 1.19–2.76), the LMR (HR = 0.52, 95% CI: 0.35–0.79) and fibrinogen (HR = 2.24, 95% CI: 1.41–3.55) were independent significant prognostic factors (Table 4). IL-8 (HR = 2.33, 95% CI: 1.32–4.11) and the LMR (HR = 0.41, 95% CI: 0.24–0.67) were independently and significantly associated with OS.

**Table 2:** Profiles of cytokine and systemic inflammatory markers.

	Median	IQR	Mean	SD
IL-1β, pg/ml	2.47	1.94–3.28	2.84	1.42
IL-6, pg/ml	5.34	3.93–7.18	5.86	3.04
IL-8, pg/ml	4.17	2.81–8.72	7.17	9.23
IL-9, pg/ml	0.40	0.07–1.00	0.87	1.55
IL-10, pg/ml	1.69	1.46–2.01	1.87	1.26
TNF-α, pg/ml	8.15	5.87–11.8	9.94	9.67
VEGF-A, pg/ml	0.62	0.04–0.93	0.78	0.97
NLR	1.81	1.31–2.76	2.29	1.66
PLR	141.51	108.46–195.92	161.69	79.33
LMR	3.70	2.62–5.00	3.97	1.87
PNI	47.69	44.71–51.31	48.10	5.02
Fibrinogen, mg/dl	330.50	284.00–397.25	350.38	95.02

*IL* interleukin, *TNF* tumour necrosis factor, *VEGF* vascular endothelial growth factor, *NLR* neutrophil-to-lymphocyte ratio, *PLR* platelet-to-lymphocyte ratio, *LMR* lymphocyte-to-monocyte ratio, *PNI* prognostic nutritional index.

**Table 3:** Univariate analysis for progression-free and overall survival.

Variable	*N*	5YR-PFS	*P* value	5YR-OS	*P* value
*Age*			<0.001		<0.001
≤60 years	186	78.9%		91.0%	
>60 years	214	65.3%		76.9%	
*Gender*			0.289		0.310
Male	244	70.6%		82.8%	
Female	156	73.5%		84.6%	
Body mass index*			0.685		0.686
≤25 kg/m^2^	279	72.7%		83.1%	
>25 kg/m^2^	120	69.4%		84.4%	
*Location*			0.055		0.187
Colon	211	78.0%		85.6%	
Rectum	189	67.3%		81.2%	
*Serum CEA*			<0.001		0.001
≤5 ng/mL	261	78.6%		88.5%	
>5 ng/mL	139	58.9%		74.2%	
*Histologic grade*^†^			0.255		0.003
Low	376	72.5%		85.1%	
High	22	60.2%		59.8%	
*Stage*			<0.001		<0.001
1	105	89.8%		93.8%	
2	110	79.4%		91.9%	
3	149	67.1%		82.3%	
4	36	16.7%		31.1%	
*Lymphovascular invasion*^‡^			<0.001		0.001
No	74	88.7%		95.8%	
Yes	311	66.7%		80.1%	
*Adjuvant treatment*			0.128		0.948
No	135	76.9%		84.5%	
Yes	265	69.2%		83.0%	
*IL-1β*			0.909		0.813
Low (≤2.47 pg/ml)	201	72.9%		85.4%	
High (>2.47 pg/ml)	199	70.5%		81.7%	
*IL-6*			<0.001		<0.001
Low (≤ 5.34 pg/ml)	200	82.2%		90.9%	
High (> 5.34 pg/ml)	200	61.1%		75.9%	
*IL-8*			<0.001		<0.001
Low (≤4.17 pg/ml)	201	81.7%		90.8%	
High (>4.17 pg/ml)	199	61.6%		76.2%	
*IL-9*			0.151		0.069
Low (≤0.40 pg/ml)	199	73.8%		86.2%	
High (>0.40 pg/ml)	201	69.7%		80.7%	
*IL-10*			0.682		0.607
Low (≤1.69 pg/ml)	209	69.7%		84.3%	
High (>1.69 pg/ml)	191	73.9%		82.7%	
*TNF-α*			0.205		0.431
Low (≤8.15 pg/ml)	200	70.2%		83.8%	
High (>8.15 pg/ml)	200	73.2%		83.3%	
*VEGF-A*			0.005		0.045
Low (≤0.62 pg/ml)	201	77.8%		87.2%	
High (>0.62 pg/ml)	199	65.5%		79.7%	
*mGPS*^§^			0.012		0.003
0	292	75.6%		87.5%	
1	55	58.9%		72.9%	
2	21	53.5%		62.6%	
*NLR*			0.035		0.011
≤5	380	73.0%		84.7%	
>5	19	50.4%		57.4%	
*PLR*			0.739		0.523
≤150	218	72.7%		85.1%	
>150	181	71.1%		81.7%	
*LMR*			0.008		0.003
≤3	140	64.6%		74.9%	
>3	259	75.9%		88.1%	
*PNI*			0.816		0.814
≤45	104	69.8%		81.8%	
>45	295	72.6%		84.0%	
*Fibrinogen*^‖^			<0.001		<0.001
Low (≤330.5 mg/dl)	193	83.7%		90.8%	
High (>330.5 mg/dl)	193	60.0%		75.7%	

*PFS* progression-free survival, *OS* overall survival, *CEA* carcinoembryonic antigen, *IL* interleukin, *TNF* tumour necrosis factor, *VEGF* vascular endothelial growth factor, *mGPS* modified Glasgow Prognostic Score, *NLR* neutrophil-to-lymphocyte ratio, *PLR* platelet-to-lymphocyte ratio, *LMR* lymphocyte-to-monocyte ratio, *PNI* prognostic nutritional index.

^*^399, ^†^398, ^‡^385, ^§^368 and ^‖^386 were available.

**Table 4:** Multivariable analysis for progression-free and overall survival (forward selection).

Variable	Progression-free survival*			Overall survival^†^		
	HR	95% CI	*P* value	HR	95% CI	*P* value
*Age*			<0.001			<0.001
≤60 years	Reference			Reference		
>60 years	2.22	1.46–3.36		4.56	2.51–8.26	
*Location*			0.001			
Colon	Reference					
Rectum	2.07	1.36–3.15				
*Histologic grade*						0.006
Low				Reference		
High				2.90	1.35–6.25	
*Stage*			<0.001			<0.001
1	Reference			Reference		
2	2.11	0.92–4.80	0.077	1.66	0.58–4.73	0.343
3	3.82	1.73–8.42	0.001	3.87	1.47–10.20	0.006
4	18.94	7.96–45.11	<0.001	17.25	6.26–47.57	<0.001
*Lymphovascular invasion*			0.043			
No	Reference					
Yes	2.48	1.03–5.96				
*IL-8*			0.005			0.003
Low (≤4.17 pg/ml)	Reference			Reference		
High (>4.17 pg/ml)	1.82	1.19–2.76		2.33	1.32–4.11	
*mGPS*			0.051			
0	Reference					
1	1.39	0.82–2.36	0.224			
2	0.47	0.20–1.07	0.072			
*LMR*			0.002			<0.001
≤3	Reference			Reference		
>3	0.52	0.35–0.79		0.41	0.24–0.67	
*Fibrinogen*			0.001			
Low (≤330.5 mg/dl)	Reference					
High (>330.5 mg/dl)	2.24	1.41–3.55				

*HR* hazard ratio, *IL* interleukin, *mGPS* modified Glasgow Prognostic Score, *LMR* lymphocyte-to-monocyte ratio.

*Adjusted with age, location, serum CEA, stage, lymphovascular invasion, modified Glasgow prognostic score, IL-6, IL-8, VEGF, NLR, LMR and fibrinogen.

^†^Adjusted with age, serum CEA, histologic grade, stage, lymphovascular invasion, modified Glasgow Prognostic Score, IL-6, IL-8, IL-9, VEGF, NLR, LMR and fibrinogen.

High concentration of IL-8 was associated with high CEA level. Patients with high LMR had higher body mass index and more colonic location than those with low LMR. Elevated fibrinogen levels were correlated with older age, colonic location, high CEA, high stage and lymphovascular invasion (Supplementary Table 7).

To identify the effect of tumour molecular alterations on prognosis, survival analysis according to IL-8 and LMR was performed with stratification of molecular alterations. Because *BRAF, NRAS* and *PTEN* had low rates of mutation, *KRAS, APC, PIK3CA* and *TP53* were included for survival analysis. In patients without *APC* mutation, IL-8 and LMR were significant prognostic factors. However, those were not significant in patients with *APC* mutation (Supplementary Table 8). In terms of *KRAS, PIK3CA* and *TP53* mutation status, IL-8 was a significant risk factor, regardless of mutation. On the other hand, LMR had inconsistent survival outcomes.

**Fig. 2:**
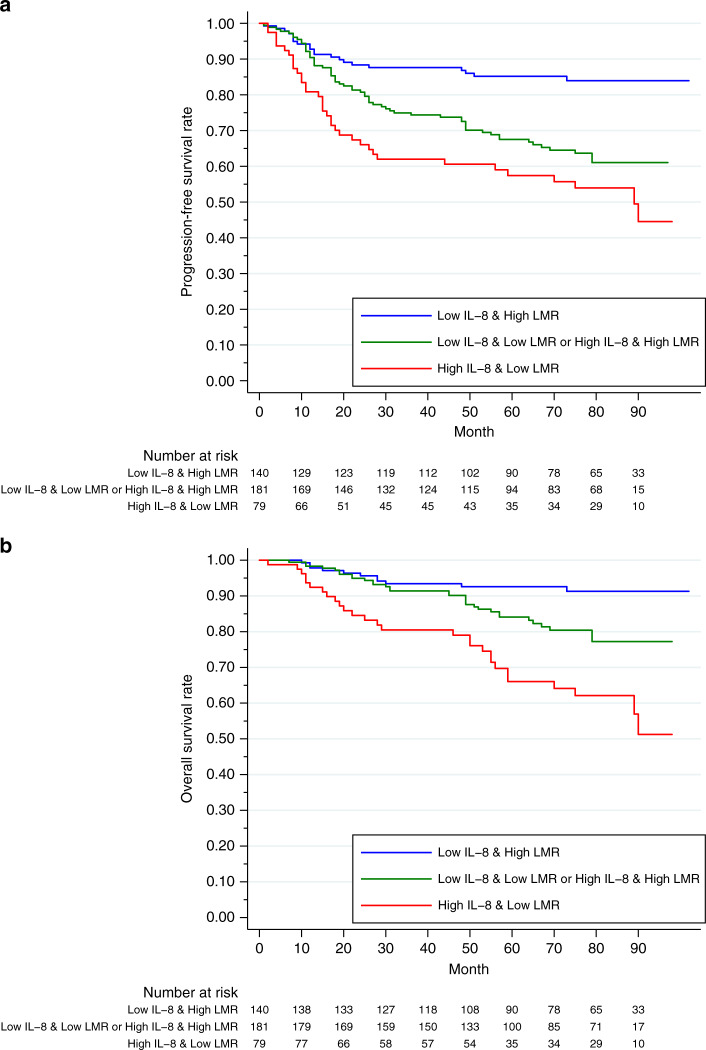
Progression-free survival (PFS) and overall survival (OS) according to the combination of interleukin (IL)-8 and the lymphocyte-to-monocyte ratio (LMR). **a** PFS (*P* < 0.001; low-risk group [low IL-8 and high LMR] vs intermediate-risk group [low IL-8 with low LMR or high IL-8 with high LMR], *P* < 0.001; low- vs high-risk group [high IL-8 and low LMR], *P* < 0.001; intermediate- vs high-risk group, *P* = 0.066). **b** OS (*P* < 0.001; low- vs intermediate-risk group, *P* = 0.008; low- vs high-risk group, *P* < 0.001; intermediate- vs high-risk group, *P* = 0.002).

IL-8 and LMR were independent, significant factors for both PFS and OS. All patients were categorised into three groups based on their IL-8 and LMR levels: a low-risk group (low IL-8 and a high LMR), an intermediate-risk group (low IL-8 with a low LMR or high IL-8 with a high LMR) and a high-risk group (high IL-8 and a low LMR). Among these three groups, both PFS and OS were significantly different (Fig. 2, *P* < 0.001, respectively). The low-risk group had the best survival, and the high-risk group had the worst survival in terms of both PFS and OS (5-year PFS: 85.2% vs 57.4%; 5-year PFS: 92.5% vs 66.1%, respectively).

## Discussion

This study focused on the interrelationships and clinical impact of serum cytokine profiles and systemic inflammatory markers in CRC. It was demonstrated that serum cytokines and systemic inflammatory markers in CRC have moderate-to-strong intragroup correlations but only relatively weak intergroup correlations.

Both serum cytokines and systemic inflammatory markers were found to be associated with survival. The combination of these two factors can aid in the survival stratification of CRC patients.

Little research has been conducted exploring the relationship between serum cytokine profiles and systemic inflammatory markers in CRC. Chen et al. reported that a high NLR correlated with a distinct cytokine profile in metastatic CRC.^26^ In this study, the correlation between the NLR and cytokines was weak (IL-1β: ρ = 0.10, IL-6: ρ = 0.17 and VEGF-A: ρ = 0.10). This difference may have been due to the different patients (metastatic vs all stages) and the type of variables (dichotomous vs continuous). Significant correlations among serum cytokine profiles and systemic inflammatory markers were evident, but the correlations between cytokines and systemic inflammatory markers were generally indistinct in this study. These results suggest that serum cytokine levels are not the only factors that influence systemic inflammatory markers. In Fig. 1, IL-6 had weak-to-strong correlations with all cytokines and systemic inflammatory markers, except the PLR. This body of work suggests that IL-6 may be regarded as a central player in the systemic inflammation of CRC.

None of the cytokines and systemic inflammatory markers was related to pathologic molecular subtypes in this study. However, some of the systemic inflammatory markers were associated with tumour somatic mutation and tumour immune score unlike cytokines (Supplementary Tables 5 and 6). These results suggest that systemic cytokines were not directly related to the characteristics of primary tumour, but some of the systemic inflammatory markers were associated with primary tumour in terms of common molecular alteration and tumour response. The association between the molecular characteristics of primary tumour and cytokines or systemic inflammatory markers is not well known. Chen et al. showed that NLR was not related with tumour molecular alteration, including *KRAS, NRAS, BRAF, PIK3CA* mutation, PTEN loss and CIMP, in metastatic CRC.^26^ However, NLR was associated with *KRAS* and *PIK3CA* mutation in this study (Supplementary Table 5). These discrepancies may be from the dissimilarity in handling of LNR as a dichotomised or continuous variable because the absolute differences of NLR values between no mutation and mutation were relatively small in this study. Another study investigated the immunologic effect of SMAD4 on tumour microenvironment of CRC.^27^ SMAD4-negative CRC had more tumour-infiltrating neutrophils with high expression of IL-8 than SMAD4-positive CRC. This result suggested that SMAD4-negative CRC may be associated with high serum IL-8 level. Further researches using multi-omics data are required to reveal the relationship between markers of inflammation and tumour intrinsic characteristics.

It is not yet clear that systemic inflammation is related with local immunity. One study demonstrated that serum cytokines and tumour-infiltrating immune cells have high intragroup correlations but only relatively weak intergroup correlations.^28^ These results were consistent with those of the present study. Another study investigated the association between local immune cell density and systemic inflammatory makers.^29^ Among systemic inflammatory markers, LMR was significantly related with the CD3 + T cells in the core of the tumour. The other study demonstrated that high NLR was related with less lymphocytic reaction at the tumour-invasive margin.^30^ In the present study, high NLR and fibrinogen level were also associated with low immune score, while PNI was positively associated with immune score (Supplementary Table 6). These results suggest that some of the systemic inflammatory markers could relate to local immunity in CRC. More studies using precise classification of immune cell categories may be needed (e.g. M1/M2 macrophage and N1/N2 neutrophil) to elucidate their relationship.

In the results of survival analysis-stratified tumour mutation, IL-8 and LMR were significant prognostic factors in patients without *APC* mutation, but not in patients with *APC* mutation. A study demonstrated that *APC* controls regulatory T-cell differentiation through microtubule-mediated nuclear factor of activated T-cell (NFAT) localisation in mouse model.^31^ *APC* mutation impairs T-cell activation and anti-inflammatory function. This impact of *APC* on the immune system and inflammation may affect the prognostic outcomes of IL-8 and LMR according to the status of *APC* mutation. Therefore, in specific tumour subtypes, cytokines and systemic inflammatory markers can be more effectively used as prognostic markers.

In the tumour microenvironment, IL-6 acts as a pleiotropic proinflammatory cytokine, which has several main roles in cancer progression, migration and angiogenesis.^32^ Circulating IL-6 is associated with tumour stage, metastasis and survival.^6^ In a meta-analysis, patients with a high serum IL-6 level had a 1.76-fold higher risk of poor OS and a 2.97-fold higher risk of poor disease-free survival.^33^ In this study, IL-6 was associated with survival in univariate but not in multivariable analysis. Because there were significant correlations within cytokines and systemic inflammatory markers, a forward selection method was used in multivariable analysis to identify more robust prognostic factors. Although IL-6 is a key mediator of tumour-associated inflammation, IL-6 was not a stronger prognostic factor than IL-8 in multivariable analysis.

IL-8 is a proinflammatory CXC chemokine that is known to have tumorigenic and proangiogenic properties.^34^ The main functions of IL-8 are the promotion of neutrophil chemotaxis and angiogenic responses in endothelial cells. IL-8 is suggested to play a multifactorial role in the angiogenesis, tumour growth, metastasis and chemoresistance of tumours in vivo.^35^ IL-8 helps to recruit myeloid-derived suppressor cells, which inhibit T-cell proliferation and activation, into the tumour microenvironment in xenograft models.^36^ IL-8 was suggested as a potential prognostic marker in CRC.^8^ In this study, high IL-8 was also independently associated with poor survival. IL-8 is correlated with tumour burden in xenograft models, and in patients with several cancer types.^37^ IL-8 could be an effective marker of early response to immunotherapy.^38^ Pharmaceutical agents inhibiting IL-8 may be a therapeutic option for targeting the tumour microenvironment. Ongoing clinical trials are evaluating the combination of anti-IL-8 antibody and immunotherapy for solid tumours.^39,40^

As a significant prognostic marker among systemic inflammatory markers in this study, the LMR consists of the lymphocyte count, which measures the degree of responsiveness of antitumour immune responses, and the monocyte count, which is an indicator of tumour proliferation.^41^ The monocyte count is supposed to reflect the formation of tumour-associated macrophages, which develop from circulating monocytes in the tumour microenvironment.^42^ A low LMR should have a low lymphocyte count or a high monocyte count. This low LMR could reflect an ineffective antitumour immune response and an elevated tumour burden. Thus, the LMR might be a strong predictor of prognosis in patients with CRC. A low LMR is associated with decreased survival in CRC.^16^

Fibrinogen was independently associated with survival in terms of only PFS. The role of fibrinogen as a promotor of tumour progression has been demonstrated in experimental studies, as it modulates angiogenesis and the metastasis of cancer cells.^43^ High-plasma fibrinogenaemia is significantly associated with worse survival in CRC.^17,44^

The mechanism explaining the association between systemic inflammation and survival outcome in CRC remains poorly understood. Tumour microenvironment may be one of the major factors in the mechanism. In tumour microenvironment, tumour-associated macrophages are derived and differentiated from circulating monocytes. Recent work has highlighted that tumour-associated macrophages enhance tumour progression and increase monocyte infiltration into the tumour site by CCL8.^45^ Another study has reported that soluble factors released by CRC cells can change tumour-specific genetic signatures in circulating monocytes.^46^ Tumour necrosis can provoke systemic inflammatory response, and represent poor outcomes in CRC.^47^ Gurthrie and co-workers found that tumour necrosis is related with IL-6, IL-10, VEGF and mGPS.^48^ In these ways, systemic inflammation may be connected with tumour microenvironment.

In stage II CRC, to identify a high-risk group is clinically meaningful for the use of adjuvant treatment. IL-8, LMR and risk groups based on their IL-8 and LMR levels were investigated as prognostic factors in stage II patients. High IL-8 was associated with poor PFS (*P* = 0.040). Low LMR was related with poor OS (*P* = 0.015). The low-risk group based on their IL-8 and LMR levels had better OS than the high-risk group (*P* = 0.025), and PFS than the intermediate-risk group (*P* = 0.048). Studies with larger sample sizes will be needed to examine these markers as prognostic factors in stage II CRC.

This study shows that the survival of CRC is not only determined by tumour characteristics, but also by inflammation and interactions between the tumour and the host. Systemic cytokine profiles and inflammatory markers, together with the current pathologic staging, provide complimentary predictive information for CRC.

To the best of our knowledge, this study was the first to investigate the relationship between serum cytokines and systemic inflammatory markers in CRC at all stages. The limitation of this study was that it assessed only seven cytokine markers. Future robust studies with many cytokine markers will reveal more information on the relationship between serum cytokines and systemic inflammatory markers. Another limitation was the inclusion of patients with neoadjuvant chemoradiotherapy. These patients may have different cytokine and systemic inflammatory profiles after neoadjuvant treatment. Although relatively small portions of patients (about 10%) were treated with neoadjuvant treatment, this treatment can be a potential confounder.

In conclusion, the studied systemic cytokine profiles and inflammatory markers have relatively weak intergroup correlations. A composite classification of systemic cytokine profiles and inflammatory markers has increased prognostic value in CRC, specifically LMR and IL-8 values.

## Supplementary information

Supplementary File

## Data Availability

The data sets used and analysed during this study are available from the corresponding author on reasonable request.
