# Supplementary material for: The relationships between systemic cytokine profiles and inflammatory markers in colorectal cancer and the prognostic significance of these parameters
Source: Br J Cancer. 2020 Jun 3;123(4):610–8. doi: 10.1038/s41416-020-0924-5 (PMC7435263; doi:10.1038/s41416-020-0924-5)
Supplement: Supplementary file 1 — Supplementary File [file 41416_2020_924_MOESM1_ESM.pdf]

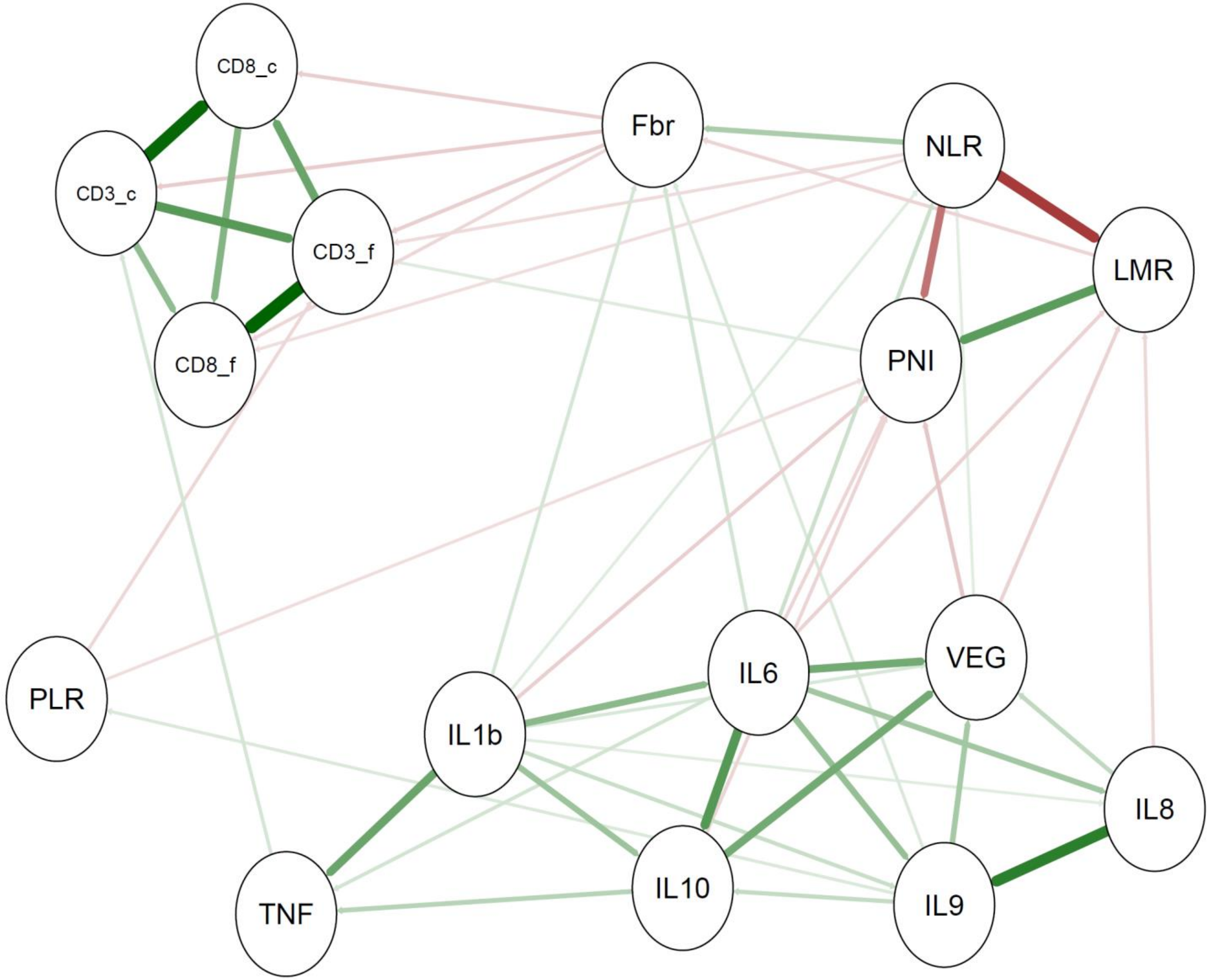
CD8_c
CD3_c
CD3_f
CD8_f
Fbr
NLR
LMR
PNI
PLR
IL1b
IL6
VEG
IL8
TNF
IL10
IL9

**Supplementary Figure 1. Pairwise correlation graph among tumour infiltrating lymphocyte and systemic cytokine profiles and systemic inflammatory markers.** Nodes represent systemic cytokine profiles or systemic inflammatory markers, and lines represent their pairwise correlations. Only significant ($p < 0.05$) correlations are shown. Line thickness indicates the strength of Spearman's correlation. Green represents positive correlations, and red represents negative correlations. CD3_c, $CD3^{+}$ T cell count in the centre of tumour; CD3_f, $CD3^{+}$ T cell count in the front of tumour; CD8_c, $CD8^{+}$ T cell count in the centre of tumour; CD8_f, $CD8^{+}$ T cell count in the front of tumour; IL, interleukin; TNF, tumour necrosis factor; VEGF, vascular endothelial growth factor; NLR, neutrophil-to-lymphocyte ratio; PLR, platelet-to-lymphocyte ratio; LMR, lymphocyte-to-monocyte ratio; PNI, prognostic nutritional index; and Fbr, fibrinogen.

**Supplementary Table 1. Somatic Mutation Panel for Colorectal Cancer**

| Assay ID | Mutation Targeted |
|---|---|
| *APC*_1 | R876* |
| *APC*_2 | R1114* |
| *APC*_3 | E1309fs*4 c.3927_3931delAAAGA#p.E1309fs*4 |
| *APC*_4 | Q1338* |
| *APC*_5 | Q1429* |
| *APC*_6 | S1465fs*3 c.4392_4393delGA#p.S1465fs*3 |
| *APC*_7 | T1661fs*9 |
| *APC*_8 | E1309fs*4 c.3922_3926delAAAGA#p.E1309fs*4 |
| *APC*_9 | S1465fs*3 c.4386_4387delGA#p.S1465fs*3 |
| *BRAF*_1 | D594V |
| *BRAF*_2 | V600E/V600R |
| *BRAF*_3 | V600K/V600L/V600R |
| *HRAS*_1 | Q61L/Q61R/Q61P |
| *KRAS*_1 | A59T |
| *KRAS*_2 | G12A/G12D/G12V |
| *KRAS*_3 | G12C/G12R/G12S |
| *KRAS*_4 | G13D |
| *KRAS*_5 | Q61H |
| *KRAS*_6 | Q61L |
| *MET*_1 | R970C |
| *MET*_2 | T992I |
| *NRAS*_1 | G12C |
| *NRAS*_2 | G12V |
| *NRAS*_3 | G13C |

| | |
|---|---|
| *NRAS*_4 | G13V |
| *NRAS*_5 | Q61E |
| *NRAS*_6 | Q61H |
| *PIK3CA*_1 | C420R |
| *PIK3CA*_2 | E542K |
| *PIK3CA*_3 | E545K |
| *PIK3CA*_4 | H701P |
| *PIK3CA*_5 | H1047R |
| *PIK3CA*_6 | Q546K |
| *PIK3CA*_7 | R88Q |
| *PTEN*_1 | R233* |
| *TP53*_1 | V157F |
| *TP53*_2 | R158C/R158G |
| *TP53*_3 | R158L/R158P |
| *TP53*_4 | Y163C |
| *TP53*_5 | R175L/R175H |
| *TP53*_6 | Y220C |
| *TP53*_7 | G245S/G245C |
| *TP53*_8 | G245D/G245V |
| *TP53*_11 | R248W/R248G |
| *TP53*_12 | R248L/R248Q |
| *TP53*_13 | R249S |
| *TP53*_14 | R249W |
| *TP53*_15 | R249M |
| *TP53*_16 | R273C |
| *TP53*_17 | R273H/R273L/R273P |
| *TP53*_18 | R282G/R282W |

**Supplementary Table 2. The REMARK checklist**

| | Item to be reported | Section |
|---|---|---|
| **INTRODUCTION** | | |
| 1 | State the marker examined, the study objectives, and any pre-specified hypotheses. | Background (page no. 5) |
| **MATERIALS AND METHODS** | | |
| *Patients* | | |
| 2 | Describe the characteristics (e.g., disease stage or co-morbidities) of the study patients, including their source and inclusion and exclusion criteria. | Methods (page no. 5) |
| 3 | Describe treatments received and how chosen (e.g., randomized or rule-based). | Methods (page no. 5,9) |
| *Specimen characteristics* | | |
| 4 | Describe type of biological material used (including control samples) and methods of preservation and storage. | Methods (page no. 5) |
| *Assay methods* | | |
| 5 | Specify the assay method used and provide (or reference) a detailed protocol, including specific reagents or kits used, quality control procedures, reproducibility assessments, quantitation methods, and scoring and reporting protocols. Specify whether and how assays were performed blinded to the study endpoint. | Methods (page no. 5,6) |
| *Study design* | | |
| 6 | State the method of case selection, including whether prospective or retrospective and whether stratification or matching (e.g., by stage of disease or age) was used. Specify the time period from which cases were taken, the end of the follow-up period, and the median follow-up time. | Methods (page no. 5) |
| 7 | Precisely define all clinical endpoints examined. | Methods (page no. 10) |
| 8 | List all candidate variables initially examined or considered for inclusion in models. | Methods (page no. 5,6) |
| 9 | Give rationale for sample size; if the study was designed to detect a specified effect size, give the target power and effect size. | Methods (page no. 10) |
| *Statistical analysis methods* | | |

| | | |
|---|---|---|
| 10 | Specify all statistical methods, including details of any variable selection procedures and other model-building issues, how model assumptions were verified, and how missing data were handled. | Methods (page no. 10) |
| 11 | Clarify how marker values were handled in the analyses; if relevant, describe methods used for cutpoint determination. | Methods (page no. 10) |
| **RESULTS** | | |
| *Data* | | |
| 12 | Describe the flow of patients through the study, including the number of patients included in each stage of the analysis (a diagram may be helpful) and reasons for dropout. Specifically, both overall and for each subgroup extensively examined report the numbers of patients and the number of events. | Results (page no. 10) |
| 13 | Report distributions of basic demographic characteristics (at least age and sex), standard (disease-specific) prognostic variables, and tumor marker, including numbers of missing values. | Results (page no. 10) Table 1 |
| *Analysis and presentation* | | |
| 14 | Show the relation of the marker to standard prognostic variables. | Results (page no. 12) Supplementary Table 7 |
| 15 | Present univariable analyses showing the relation between the marker and outcome, with the estimated effect (e.g., hazard ratio and survival probability). Preferably provide similar analyses for all other variables being analyzed. For the effect of a tumor marker on a time-to-event outcome, a Kaplan-Meier plot is recommended. | Table 3 |
| 16 | For key multivariable analyses, report estimated effects (e.g., hazard ratio) with confidence intervals for the marker and, at least for the final model, all other variables in the model. | Table 4 |
| 17 | Among reported results, provide estimated effects with confidence intervals from an analysis in which the marker and standard prognostic variables are included, regardless of their statistical significance. | Results (page no. 11) |
| 18 | If done, report results of further investigations, such as checking assumptions, sensitivity analyses, and internal validation. | Not done |
| **DISCUSSION** | | |
| 19 | Interpret the results in the context of the pre-specified hypotheses and other relevant studies; include a discussion of limitations of the study. | Discussion (page no. 13-15) |
| 20 | Discuss implications for future research and clinical value. | Discussion (page no. 15) |

**Supplementary Table 3. Spearman's rho correlation coefficients between cytokines and systemic inflammatory markers**

| | IL-1b | IL-6 | IL-8 | IL-9 | IL10 | TNF-a | VEGF | NLR | PLR | LMR | PNI | Fibrinogen |
|---|---|---|---|---|---|---|---|---|---|---|---|---|
| IL-1b | 1 | 0.39** | 0.10* | 0.18** | 0.33** | 0.50** | 0.13** | 0.10* | 0.09 | -0.07 | -0.16** | 0.14** |
| IL-6 | | 1 | 0.31** | 0.35** | 0.57** | 0.15** | 0.47** | 0.17** | 0.07 | -0.15* | -0.14** | 0.15** |
| IL-8 | | | 1 | 0.71** | 0.09 | 0.02 | 0.21** | 0.07 | 0.03 | -0.13* | -0.06 | 0.07 |
| IL-9 | | | | 1 | 0.20** | 0.07 | 0.30** | 0.03 | 0.12* | -0.08 | -0.07 | 0.12* |
| IL10 | | | | | 1 | 0.24** | 0.47** | 0.07 | 0.05 | -0.07 | -0.14** | 0.01 |
| TNF-a | | | | | | 1 | 0.03 | -0.01 | -0.03 | 0.05 | 0.01 | 0.08 |
| VEGF | | | | | | | 1 | 0.10** | 0.08 | -0.15* | -0.19** | 0.01 |
| NLR | | | | | | | | 1 | 0.07 | -0.66** | -0.47** | 0.28** |
| PLR | | | | | | | | | 1 | -0.09 | -0.11** | 0.07 |
| LMR | | | | | | | | | | 1 | 0.55** | -0.14** |
| PNI | | | | | | | | | | | 1 | -0.08 |
| Fibrinogen | | | | | | | | | | | | 1 |

** Correlation is significant at the 0.01 level (2-tailed)

* Correlation is significant at the 0.05 level (2-tailed)

IL, interleukin; TNF, tumor necrosis factor; VEGF, vascular endothelial growth factor, NLR: Neutrophil-Lymphocyte ratio; PLR: Platelet-Lymphocyte ratio; LMR: Lymphocyte-Monocyte ratio; PNI: Prognostic nutrition index

**Supplementary Table 4. Cytokines and systemic inflammatory markers according to pathologic molecular subtype**

| | Immune (CMS1) (N= 28) | Epithelial (CMS2/3) (N=87) | Mesenchymal (CMS4) (N= 23) | p-value |
|---|---|---|---|---|
| IL-1β, pg/ml | 1.96 ± 1.95 | 2.93 ± 1.1 | 2.8 ± 1.34 | 0.864 |
| IL-6, pg/ml | 2.85 ± 1.32 | 6.55 ± 6 | 5.89 ± 3.8 | 0.724 |
| IL-8, pg/ml | 6 ± 4.09 | 5.43 ± 5.71 | 7.07 ± 11.79 | 0.675 |
| IL-9, pg/ml | 0.52 ± 0.96 | 0.77 ± 1.49 | 0.62 ± 0.7 | 0.638 |
| IL-10, pg/ml | 2.73 ± 4.14 | 1.76 ± 0.63 | 1.77 ± 0.41 | 0.066 |
| TNF-α, pg/ml | 11.1 ± 6.79 | 9.06 ± 5.28 | 8.79 ± 5.04 | 0.206 |
| VEGF-A, pg/ml | 1.04 ± 1.86 | 0.71 ± 0.75 | 0.52 ± 0.36 | 0.19 |
| NLR | 2.16 ± 1.81 | 2.12 ± 1.36 | 2.08 ± 0.7 | 0.983 |
| PLR | 134.86 ± 47.12 | 155.85 ± 84.73 | 167.98 ± 54.99 | 0.258 |
| LMR | 3.99 ± 1.35 | 4.22 ± 1.68 | 4 ± 1.67 | 0.724 |
| PNI | 49.27 ± 6.28 | 49.08 ± 4.51 | 48.03 ± 3.51 | 0.594 |
| Fibrinogen, mg/dl | 337.39 ± 82.93 | 365.26 ± 105.14 | 369.36 ± 82.69 | 0.38 |

Mean ± standard deviation, CMS, consensus molecular subtype; IL, interleukin; TNF, tumor necrosis factor; VEGF, vascular endothelial growth factor; NLR, neutrophil to lymphocyte ratio; PLR, platelet to lymphocyte ratio; LMR, lymphocyte to monocyte ratio; PNI, prognostic nutritional index

**Supplementary Table 5. Cytokines and systemic inflammatory markers according to tumour molecular alteration (1)**

| | *KRAS* No mutation (N=215) | *KRAS* Mutation (N=114) | p-value | *BRAF* No mutation (N=326) | *BRAF* Mutation (N=2) | p-value |
|---|---|---|---|---|---|---|
| IL-1β, pg/ml | 2.95 ± 1.54 | 2.94 ± 1.4 | 0.915 | 2.95 ± 1.49 | 2.07 ± 0.65 | 0.298 |
| IL-6, pg/ml | 6.11 ± 3.67 | 5.64 ± 2.16 | 0.381 | 5.96 ± 3.25 | 5.28 ± 0.1 | 0.858 |
| IL-8, pg/ml | 7.38 ± 9.18 | 7.09 ± 11.22 | 0.863 | 7.28 ± 9.95 | 9.11 ± 7.94 | 0.402 |
| IL-9, pg/ml | 0.85 ± 1.58 | 0.85 ± 1.73 | 0.701 | 0.85 ± 1.64 | 0.38 ± 0.47 | 0.719 |
| IL-10, pg/ml | 1.95 ± 1.66 | 1.8 ± 0.53 | 0.973 | 1.9 ± 1.38 | 1.73 ± 0.06 | 0.946 |
| TNF-α, pg/ml | 10.67 ± 12.2 | 9.49 ± 5.59 | 0.784 | 10.3 ± 10.44 | 4.49 ± 2.04 | 0.80 |
| VEGF-A, pg/ml | 0.78 ± 1.05 | 0.64 ± 0.54 | 0.654 | 0.74 ± 0.91 | 0.46 ± 0 | 0.369 |
| NLR | 2.41 ± 1.79 | 2 ± 1.23 | 0.038 | 2.27 ± 1.63 | 1.28 ± 0.29 | 0.179 |
| PLR | 166.12 ± 80.54 | 148.2 ± 64.88 | 0.067 | 160.15 ± 76.14 | 124.24 ± 11.21 | 0.514 |
| LMR | 3.67 ± 1.52 | 4.49 ± 2.08 | 0.001 | 3.95 ± 1.78 | 3.94 ± 0.83 | 0.857 |
| PNI | 47.74 ± 5.05 | 49.07 ± 4.78 | 0.002 | 48.2 ± 5.01 | 50.47 ± 2.66 | 0.380 |
| Fibrinogen, mg/dl | 356.78 ± 96.13 | 360.07 ± 97.31 | 0.625 | 357.95 ± 96.7 | 351.5 ± 16.26 | 0.895 |

Mean ± standard deviation, IL, interleukin; TNF, tumor necrosis factor; VEGF, vascular endothelial growth factor; NLR, neutrophil to lymphocyte ratio; PLR, platelet to lymphocyte ratio; LMR, lymphocyte to monocyte ratio; PNI, prognostic nutritional index

**Supplementary Table 5. Cytokines and systemic inflammatory markers according to tumour molecular alteration (2)**

| | *NRAS* No mutation (N=326) | *NRAS* Mutation (N=2) | p-value | *APC* No mutation (N=280) | *APC* Mutation (N=41) | p-value |
|---|---|---|---|---|---|---|
| IL-1β, pg/ml | 2.95 ± 1.49 | 2.78 ± 0.98 | 0.917 | 2.97 ± 1.51 | 2.8 ± 1.46 | 0.364 |
| IL-6, pg/ml | 5.96 ± 3.25 | 4.55 ± 1.55 | 0.373 | 5.91 ± 2.91 | 6.48 ± 5.09 | 0.719 |
| IL-8, pg/ml | 7.3 ± 9.96 | 5.33 ± 1.73 | 0.731 | 7.01 ± 9.17 | 9.66 ± 14.53 | 0.534 |
| IL-9, pg/ml | 0.85 ± 1.64 | 1.23 ± 0.98 | 0.213 | 0.8 ± 1.48 | 1.29 ± 2.51 | 0.050 |
| IL-10, pg/ml | 1.9 ± 1.38 | 1.62 ± 0.78 | 0.745 | 1.89 ± 1.45 | 1.87 ± 0.83 | 0.804 |
| TNF-α, pg/ml | 10.27 ± 10.44 | 10.11 ± 5.28 | 0.802 | 10.3 ± 10.98 | 9.61 ± 5.87 | 0.993 |
| VEGF-A, pg/ml | 0.74 ± 0.91 | 0.3 ± 0.37 | 0.279 | 0.72 ± 0.93 | 0.8 ± 0.8 | 0.556 |
| NLR | 2.26 ± 1.63 | 2.93 ± 1.29 | 0.224 | 2.26 ± 1.57 | 2.38 ± 2.05 | 0.378 |
| PLR | 159.9 ± 76.14 | 163.32 ± 52.63 | 0.696 | 162.08 ± 78.09 | 150.16 ± 61.14 | 0.530 |
| LMR | 3.96 ± 1.78 | 3.44 ± 0.39 | 0.744 | 3.94 ± 1.76 | 4.08 ± 1.98 | 0.785 |
| PNI | 48.22 ± 5 | 45.78 ± 4.07 | 0.431 | 48.28 ± 5.04 | 48.03 ± 4.97 | 0.907 |
| Fibrinogen, mg/dl | 357.82 ± 96.69 | 373 ± 4.24 | 0.551 | 359.64 ± 96.06 | 358.13 ± 100.65 | 0.962 |

Mean ± standard deviation, IL, interleukin; TNF, tumor necrosis factor; VEGF, vascular endothelial growth factor; NLR, neutrophil to lymphocyte ratio; PLR, platelet to lymphocyte ratio; LMR, lymphocyte to monocyte ratio; PNI, prognostic nutritional index

**Supplementary Table 5. Cytokines and systemic inflammatory markers according to tumour molecular alteration (3)**

| | *PIK3CA* No mutation (N=298) | *PIK3CA* Mutation (N=23) | p-value | *PTEN* No mutation (N=319) | *PTEN* Mutation (N=2) | p-value |
|---|---|---|---|---|---|---|
| IL-1β, pg/ml | 2.9 ± 1.37 | 3.57 ± 2.61 | 0.377 | 2.94 ± 1.5 | 3.58 ± 2.31 | 0.680 |
| IL-6, pg/ml | 6 ± 3.32 | 5.73 ± 2.5 | 0.518 | 5.97 ± 3.25 | 8.16 ± 6.21 | 0.671 |
| IL-8, pg/ml | 7.56 ± 10.33 | 4.65 ± 3.64 | 0.103 | 7.36 ± 10.05 | 5.92 ± 4.85 | 0.872 |
| IL-9, pg/ml | 0.88 ± 1.7 | 0.63 ± 0.66 | 0.861 | 0.86 ± 1.65 | 0.73 ± 1.03 | 0.730 |
| IL-10, pg/ml | 1.88 ± 1.39 | 2.09 ± 1.35 | 0.820 | 1.89 ± 1.39 | 1.64 ± 0.66 | 0.731 |
| TNF-α, pg/ml | 10.15 ± 10.69 | 11.01 ± 6.86 | 0.308 | 10.17 ± 10.45 | 17.24 ± 14.94 | 0.494 |
| VEGF-A, pg/ml | 0.72 ± 0.88 | 0.91 ± 1.27 | 0.731 | 0.74 ± 0.92 | 0.42 ± 0.54 | 0.630 |
| NLR | 2.24 ± 1.66 | 2.66 ± 1.3 | 0.017 | 2.27 ± 1.64 | 1.63 ± 0.04 | 0.673 |
| PLR | 157.26 ± 76.09 | 203.58 ± 64.8 | <0.001 | 160.81 ± 76.35 | 124.66 ± 36.36 | 0.520 |
| LMR | 3.95 ± 1.79 | 4.02 ± 1.77 | 0.890 | 3.95 ± 1.79 | 4.75 ± 0.92 | 0.326 |
| PNI | 48.32 ± 5.03 | 47.35 ± 4.96 | 0.489 | 48.23 ± 5.02 | 49.94 ± 6.4 | 0.657 |
| Fibrinogen, mg/dl | 358.84 ± 97.24 | 367.81 ± 87.06 | 0.272 | 359.79 ± 96.7 | 307.5 ± 2.12 | 0.393 |

Mean ± standard deviation, IL, interleukin; TNF, tumor necrosis factor; VEGF, vascular endothelial growth factor; NLR, neutrophil to lymphocyte ratio; PLR, platelet to lymphocyte ratio; LMR, lymphocyte to monocyte ratio; PNI, prognostic nutritional index

**Supplementary Table 5. Cytokines and systemic inflammatory markers according to tumour molecular alteration (4)**

| | *TP53* No mutation (N=224) | *TP53* Mutation (N=104) | p-value |
|---|---|---|---|
| IL-1β, pg/ml | 2.96 ± 1.5 | 2.91 ± 1.48 | 0.550 |
| IL-6, pg/ml | 6.13 ± 3.67 | 5.57 ± 1.98 | 0.199 |
| IL-8, pg/ml | 7.49 ± 9.37 | 6.86 ± 11.08 | 0.175 |
| IL-9, pg/ml | 0.91 ± 1.77 | 0.72 ± 1.29 | 0.495 |
| IL-10, pg/ml | 1.95 ± 1.61 | 1.8 ± 0.61 | 0.529 |
| TNF-α, pg/ml | 10.57 ± 11.68 | 9.59 ± 6.95 | 0.190 |
| VEGF-A, pg/ml | 0.78 ± 1.01 | 0.64 ± 0.63 | 0.317 |
| NLR | 2.37 ± 1.72 | 2.05 ± 1.38 | 0.054 |
| PLR | 166.84 ± 78.14 | 145.24 ± 68.91 | 0.014 |
| LMR | 3.82 ± 1.8 | 4.21 ± 1.67 | 0.025 |
| PNI | 47.74 ± 5.15 | 49.21 ± 4.51 | 0.005 |
| Fibrinogen, mg/dl | 359.34 ± 92.92 | 355.65 ± 104.31 | 0.398 |

Mean ± standard deviation, IL, interleukin; TNF, tumor necrosis factor; VEGF, vascular endothelial growth factor; NLR, neutrophil to lymphocyte ratio; PLR, platelet to lymphocyte ratio; LMR, lymphocyte to monocyte ratio; PNI, prognostic nutritional index

**Supplementary Table 6. Cytokines and systemic inflammatory markers according to immune score and microsatellite instability**

| | Low immune score (N=221) | High immune score (N=90) | p-value | Microsatellite instable (N=33) | Microsatellite stable (N=272) | p-value |
|---|---|---|---|---|---|---|
| IL-1β, pg/ml | 3.00 ± 1.65 | 2.87 ± 1.06 | 0.487 | 2.94 ± 1.55 | 2.98 ± 1.52 | 0.889 |
| IL-6, pg/ml | 6.03 ± 3.09 | 5.87 ± 3.78 | 0.213 | 6.44 ± 5.62 | 5.96 ± 2.94 | 0.58 |
| IL-8, pg/ml | 7.65 ± 10.43 | 6.42 ± 7.83 | 0.259 | 8.18 ± 8.92 | 7.06 ± 9.51 | 0.512 |
| IL-9, pg/ml | 0.92 ± 1.81 | 0.70 ± 1.07 | 0.707 | 0.92 ± 1.31 | 0.83 ± 1.62 | 0.548 |
| IL-10, pg/ml | 1.82 ± 0.72 | 2.13 ± 2.35 | 0.057 | 2.43 ± 3.82 | 1.85 ± 0.71 | 0.639 |
| TNF-α, pg/ml | 9.61 ± 6.4 | 11.95 ± 16.9 | 0.069 | 9.66 ± 4.49 | 10.38 ± 11.22 | 0.611 |
| VEGF-A, pg/ml | 0.70 ± 0.71 | 0.81 ± 1.17 | 0.721 | 1.08 ± 1.97 | 0.71 ± 0.71 | 0.58 |
| NLR | 2.38 ± 1.61 | 1.90 ± 1.28 | 0.005 | 2.37 ± 1.01 | 2.24 ± 1.73 | 0.033 |
| PLR | 163.51 ± 79.04 | 143.15 ± 57.69 | 0.063 | 177.5 ± 70.83 | 154.16 ± 73.95 | 0.041 |
| LMR | 3.91 ± 1.8 | 4.21 ± 1.73 | 0.093 | 3.72 ± 1.8 | 4.09 ± 1.81 | 0.152 |
| PNI | 47.96 ± 4.74 | 49.24 ± 5.12 | 0.042 | 46.59 ± 5.34 | 48.54 ± 4.79 | 0.015 |
| Fibrinogen, mg/dl | 368.19 ± 98.82 | 339.7 ± 84.97 | 0.011 | 380.41 ± 97.06 | 358.42 ± 96.53 | 0.123 |

Mean ± standard deviation, IL, interleukin; TNF, tumor necrosis factor; VEGF, vascular endothelial growth factor; NLR, neutrophil to lymphocyte ratio; PLR, platelet to lymphocyte ratio; LMR, lymphocyte to monocyte ratio; PNI, prognostic nutritional index

**Supplementary Table 7. Clinicopathologic characteristics according to interleukin-8, lymphocyte to monocyte ratio and fibrinogen**

| **Variable** | IL-8 Low (N=201) | IL-8 High (N=199) | p-value | LMR Low (N=140) | LMR High (N=259) | p-value | Fibrinogen Low (N=193) | Fibrinogen High (N=193) | p-value |
|---|---|---|---|---|---|---|---|---|---|
| Age | | | 0.611 | | | 0.39 | | | 0.002 |
| ≤ 60 years | 96 (47.8%) | 90 (45.2%) | | 69 (49.3%) | 116 (44.8%) | | 104 (53.9%) | 74 (38.3%) | |
| > 60 years | 105 (52.2%) | 109 (54.8%) | | 71 (50.7%) | 143 (55.2%) | | 89 (46.1%) | 119 (61.7%) | |
| Gender | | | 0.19 | | | 0.169 | | | 1.000 |
| Male | 129 (64.2%) | 115 (57.8%) | | 92 (65.7%) | 152 (58.7%) | | 117 (60.6%) | 117 (60.6%) | |
| Female | 72 (35.8%) | 84 (42.2%) | | 48 (34.3%) | 107 (41.3%) | | 76 (39.4%) | 76 (39.4%) | |
| Body mass index | | | 0.492 | | | 0.006 | | | 0.456 |
| ≤ 25 kg/m$^2$ | 143 (71.5%) | 136 (68.3%) | | 109 (78.4%) | 169 (65.3%) | | 132 (68.4%) | 138 (71.9%) | |
| > 25 kg/m$^2$ | 57 (28.5%) | 63 (31.7%) | | 30 (21.6%) | 90 (34.7%) | | 61 (31.6%) | 54 (28.1%) | |
| Location | | | 0.319 | | | 0.003 | | | 0.004 |
| Colon | 111 (55.2%) | 100 (50.3%) | | 60 (42.9%) | 151 (58.3%) | | 88 (45.6%) | 116 (60.1%) | |
| Rectum | 90 (44.8%) | 99 (49.7%) | | 80 (57.1%) | 108 (41.7%) | | 105 (54.4%) | 77 (39.9%) | |
| Serum CEA | | | 0.002 | | | 0.07 | | | 0.004 |
| ≤ 5 ng/mL | 146 (72.6%) | 115 (57.8%) | | 83 (59.3%) | 177 (68.3%) | | 141 (73.1%) | 114 (59.1%) | |
| > 5 ng/mL | 55 (27.4%) | 84 (42.2%) | | 57 (40.7%) | 82 (31.7%) | | 52 (26.9%) | 79 (40.9%) | |
| Histologic grade | | | 0.626 | | | 0.569 | | | 0.083 |

| | | | | | | | | | |
|---|---|---|---|---|---|---|---|---|---|
| Low | 191 (95%) | 185 (93.9%) | | 131 (93.6%) | 244 (94.9%) | | 184 (96.3%) | 178 (92.2%) | |
| High | 10 (5%) | 12 (6.1%) | | 9 (6.4%) | 13 (5.1%) | | 7 (3.7%) | 15 (7.8%) | |
| Stage | | | 0.219 | | | 0.534 | | | <0.001 |
| 1 | 59 (29.4%) | 46 (23.1%) | | 37 (26.4%) | 68 (26.3%) | | 78 (40.4%) | 25 (13%) | |
| 2 | 54 (26.9%) | 56 (28.1%) | | 42 (30%) | 68 (26.3%) | | 45 (23.3%) | 61 (31.6%) | |
| 3 | 75 (37.3%) | 74 (37.2%) | | 46 (32.9%) | 102 (39.4%) | | 59 (30.6%) | 84 (43.5%) | |
| 4 | 13 (6.5%) | 23 (11.6%) | | 15 (10.7%) | 21 (8.1%) | | 11 (5.7%) | 23 (11.9%) | |
| Lymphovascular invasion | | | 0.483 | | | 0.177 | | | <0.001 |
| No | 40 (20.6%) | 34 (17.8%) | | 31 (23%) | 43 (17.3%) | | 51 (28.2%) | 23 (12%) | |
| Yes | 154 (79.4%) | 157 (82.2%) | | 104 (77%) | 206 (82.7%) | | 130 (71.8%) | 168 (88%) | |

IL, interleukin; LMR, lymphocyte to monocyte ratio

**Supplementary Table 8. Progression-free and overall survival according to IL-8 and lymphocyte to monocyte ratio, stratified by tumor mutation (KRAS, APC, PIK3CA and TP53)**

| **Variable** | **N** | **5YR-PFS** | **p-value** | **5YR-OS** | **p-value** |
|---|---|---|---|---|---|
| KRAS – no mutation | | | | | |
| IL-8 | | | <0.001 | | 0.001 |
| Low (≤ 4.17 pg/ml) | 112 | 78.6% | | 89.2% | |
| High (> 4.17 pg/ml) | 103 | 56.3% | | 72.2% | |
| LMR | | | 0.089 | | 0.126 |
| ≤ 3 | 82 | 60.0% | | 73.3% | |
| > 3 | 132 | 73.4% | | 85.7% | |
| KRAS – mutation | | | | | |
| IL-8 | | | 0.001 | | 0.008 |
| Low (≤ 4.17 pg/ml) | 56 | 81.1% | | 90.5% | |
| High (> 4.17 pg/ml) | 58 | 63.1% | | 74.8% | |
| LMR | | | 0.127 | | 0.002 |
| ≤ 3 | 30 | 65.2% | | 67.5% | |
| > 3 | 84 | 72.4% | | 88.2% | |

| | | | | | |
|---|---|---|---|---|---|
| APC – no mutation | | | | | |
| IL-8 | | | <0.001 | | <0.001 |
| Low (≤ 4.17 pg/ml) | 143 | 80.2% | | 90.8% | |
| High (> 4.17 pg/ml) | 137 | 57.1% | | 84.0% | |
| LMR | | | 0.002 | | 0.001 |
| ≤ 3 | 96 | 57.4% | | 69.7% | |
| > 3 | 184 | 75.0% | | 88.4% | |
| APC – mutation | | | | | |
| IL-8 | | | 0.106 | | 0.453 |
| Low (≤ 4.17 pg/ml) | 19 | 73.7% | | 78.9% | |
| High (> 4.17 pg/ml) | 22 | 52.1% | | 74.6% | |
| LMR | | | 0.152 | | 0.522 |
| ≤ 3 | 13 | 83.9% | | 83.3% | |
| > 3 | 27 | 55.6% | | 73.5% | |
| PIK3CA – no mutation | | | | | |
| IL-8 | | | <0.001 | | <0.001 |
| Low (≤ 4.17 pg/ml) | 146 | 77.9% | | 88.9% | |

| | | | | | |
|---|---|---|---|---|---|
| High (> 4.17 pg/ml) | 152 | 56.2% | | 73.5% | |
| LMR | | | 0.038 | | 0.007 |
| ≤ 3 | 102 | 59.9% | | 71.5% | |
| > 3 | 195 | 71.1% | | 86.2% | |
| PIK3CA – mutation | | | | | |
| IL-8 | | | 0.025 | | 0.025 |
| Low (≤ 4.17 pg/ml) | 16 | 92.9% | | 84.4% | |
| High (> 4.17 pg/ml) | 7 | 57.1% | | 57.1% | |
| LMR | | | 0.244 | | 0.244 |
| ≤ 3 | 7 | 68.6% | | 68.6% | |
| > 3 | 16 | 87.5% | | 87.5% | |
| TP53 – no mutation | | | | | |
| IL-8 | | | <0.001 | | <0.001 |
| Low (≤ 4.17 pg/ml) | 105 | 78.9% | | 89.2% | |
| High (> 4.17 pg/ml) | 119 | 59.1% | | 74.1% | |
| LMR | | | 0.175 | | 0.038 |
| ≤ 3 | 85 | 62.7% | | 72.1% | |

| | | | | | |
|---|---|---|---|---|---|
| > 3 | 139 | 72.0% | | 86.9% | |
| TP53 – mutation | | | | | |
| IL-8 | | | 0.002 | | 0.041 |
| Low (≤ 4.17 pg/ml) | 62 | 81.9% | | 90.1% | |
| High (> 4.17 pg/ml) | 42 | 50.3% | | 70.6% | |
| LMR | | | 0.045 | | 0.049 |
| ≤ 3 | 27 | 56.9% | | 71.2% | |
| > 3 | 76 | 74.5% | | 86.2% | |

PFS, progression-free survival; OS, overall survival; IL, interleukin; LMR, lymphocyte to monocyte ratio
